# Effectiveness and Safety of Intrathecal Morphine for Pediatric Patients Undergoing Scoliosis Surgery: A Systematic Review and Meta-Analysis

**DOI:** 10.7759/cureus.51754

**Published:** 2024-01-06

**Authors:** Kashif Daud, Sajid Wariach, Mubariz Maqsood, Mohamed Sarraj, Karim Gaber, Joycelyne Ewusie, Abdulaziz Khurshed, Waleed Kishta, Mohamed Nassef

**Affiliations:** 1 Division of Orthopaedic Surgery, McMaster University, Hamilton, CAN; 2 Department of Orthopaedic Surgery, Mansoura International Hospital, Mansoura, EGY; 3 Department of Health Research Methods, Evidence, and Impact, McMaster University, Hamilton, CAN; 4 Division of Anesthesia, McMaster University, Hamilton, CAN

**Keywords:** pain, opioids, scoliosis, spinal surgery, intrathecal morphine

## Abstract

Adolescent idiopathic scoliosis (AIS) often necessitates spinal fusion surgery in pediatric patients, posing significant challenges in postoperative pain management. Standard care involves the administration of intravenous opioids perioperatively, often requiring high doses to achieve adequate analgesia following an operation. This increases the risk of adverse events, may delay recovery and prolong hospital stay, and increases the likelihood of future abuse and dependence. In this systematic review and meta-analysis, we assess the safety and effectiveness of intrathecal morphine (ITM) in pediatric patients undergoing posterior spinal fusion. Ovid Embase and MEDLINE were searched in October 2023 for articles that directly compared ITM use with standard pain management approaches for pediatric patients undergoing posterior spinal fusion. Our primary outcome was postoperative pain scores. Secondary outcomes included opioid usage details, adverse events, and blood loss. Of the 384 unique studies identified, nine studies (one randomized control trial, one prospective review, and seven retrospective reviews) met the inclusion criteria. The total number of patients within the ITM and control groups were 1384 and 676, respectively. Meta-analysis revealed significantly lower pain scores in the ITM group (standardized mean difference (SMD): -1.30 (-2.29, -0.31); p = 0.01). Similarly, ITM patients had significantly lower opioid usage, both intraoperatively (mean difference (MD): -0.71 mg/kg (-0.99, -0.44); p < 0.00001) as well as postoperatively (SMD: -2.10 (-3.48, -0.73); p = 0.003), and significantly lower blood loss (MD: -0.88 L (-1.34, -0.43); p = 0.0001). The occurrence of adverse events was similar across both groups. Our analysis of the available data demonstrates that a low to moderate dose of ITM is a safe and effective adjunct to improve standard postoperative care without increasing the risk of respiratory depression. When compared to control, ITM patients had superior analgesia while using fewer opioids had significantly reduced intraoperative blood loss when ITM was administered before spinal fusion, and had a similar complication profile. While further studies are warranted to establish optimal dosing, these findings underscore the potential of ITM as a valuable addition to multimodal pain management.

## Introduction and background

In the pediatric population, adolescent idiopathic scoliosis (AIS) is the most common spinal deformity, with an incidence of about 1-3% in otherwise healthy children [1,2]. After an initial diagnosis of AIS, patients are presented with three main courses of action: observation, bracing, or surgical intervention [3]. Following skeletal maturity, bracing is not considered effective in preventing curve progression. As a result, if the curvature exceeds a certain severity, typically exceeding 50 degrees [3], spinal fusion surgery is recommended to prevent curve progression throughout life, which can cause back pain, respiratory and cardiovascular deterioration, and a negative self-image from disfigurement [1,3,4]. Approximately 23% of patients who undergo bracing and 22% under observation for AIS require spinal fusion [5]. This procedure often results in significant postoperative pain, making pain management a primary concern in perioperative care [1].

Currently, standard pain management for spinal fusion involves the administration of intravenous opioids intraoperatively and is often followed with opioid-based patient-controlled analgesia (PCA) postoperatively [1]. Non-opioid analgesics, such as acetaminophen, and nonsteroidal anti-inflammatory drugs (NSAIDs), such as ibuprofen, as well as non-pharmacologic options like ice and heat, are also used for analgesia to minimize opioid-related side effects [6]. Despite this, most patients require high doses of opioids to achieve adequate analgesia, increasing the risk of adverse events such as nausea, vomiting, pruritus, sedation, and respiratory depression. Together, such events delay recovery, prolong hospital stay, and are a major inconvenience for patients [1]. In the long term, excessive opioid use following spine surgery can increase the length of stay, associated hospital costs, and the likelihood of future abuse and dependency [7]. Thus, minimizing opiate usage is critical in an effective pain management strategy following scoliosis surgery.

Neuraxial administration of opioids intraoperatively has been demonstrated to be an effective adjunct to improve analgesia and reduce postoperative opioid use [8,9]. Neuraxial administration includes both intrathecal administration, an injection directly into the thecal sac in the subarachnoid space, and epidural administration, an injection into the epidural space that is in close proximity to the subarachnoid space, thereby facilitating drug diffusion into the CSF. Our study focuses on intrathecal morphine (ITM), which is 10 times more potent than epidural administration as it directly acts on the opioid receptors in the central nervous system [10]. In 1979, Wang et al. presented one of the earliest documented instances of ITM application in humans. In their study, cancer patients with back and leg pain experienced prolonged pain relief with no side effects following ITM [11]. Since the late 1980s, intrathecally administered analgesics have increasingly been utilized for persistent pain caused by various conditions [12]. When injected intrathecally, morphine, along with other water-soluble opioids, has key advantages over lipophilic opioids like fentanyl. Once injected into the aqueous CSF, the hydrophilic morphine readily disperses along the neuraxis and remains in the CSF for several hours [10], exhibiting a longer duration of action and providing efficient pain relief over a wider anatomical extent along the spinal cord. This is in contrast to lipophilic opioids, which are quicker to exit the CSF and provide a shorter duration of effective analgesia [13].

While the ability of ITM to reduce opioid demand, improve analgesia, and decrease intraoperative blood loss following spinal fusion has long been recognized [14], fear of adverse events, particularly respiratory depression, has been a barrier to its adoption in certain settings [15]. The hydrophilicity and cephalad movement of morphine through the cerebrospinal fluid is known to increase the risk of delayed respiratory depression [8]. However, this risk is considered dose-dependent; a recent systematic review and meta-analysis by Wang et al. demonstrated ITM is safe and effective in the adult population [16]. With that said, there has yet to be a systematic synthesis of data for the pediatric population to our knowledge. This gap in understanding makes its true complication profile unclear. In this systematic review and meta-analysis, we compare clinical outcomes following ITM use to standard pain management for pediatric patients undergoing posterior spinal fusion surgery.

## Review

Methods

Search Strategy and Selection

Table 1 outlines our search strategy. These terms were selected to include all of the surgical topics and key terms linked with the use of pediatric ITM injection for spinal fusion surgery. Ovid Embase and MEDLINE were used for the search (including Epub ahead of print articles). To broaden the scope of the search results, we used truncations and proximity operators linked with the search phrases. The latest search was conducted in October 2023 and key articles that had previously been identified were found in the search results, confirming the breadth and accuracy of the search strategy.

**Table 1:** Search strategy

Line #	Search terms
1	scoliosis/or idiopathic scoliosis/
2	scoliosis.mp.
3	1 or 2
4	surgery.mp.
5	spine surgery/or surgery/
6	surgical technique/
7	fusion.mp.
8	exp spine fusion/
9	4 or 5 or 6 or 7 or 8
10	3 and 9
11	narcotic analgesic/or morphine/or opiate/
12	(narcotic or morphine or opioid* or opiate*).mp.
13	11 or 12
14	exp intraspinal drug administration/
15	(intraspinal or spinal or intra-spinal or intrathecal).mp.
16	14 or 15
17	13 and 16
18	10 and 17
19	exp child/
20	(child* or infant or paediatric or pediatric or kid or toddler).mp.
21	exp adolescent/
22	19 or 20 or 21
23	18 and 22

Studies were included if they compared ITM use to standard pain management (opioids administered intravenously) in pediatric patients (≤21 years old) undergoing posterior spinal fusion surgery with instrumentation for idiopathic scoliosis. The primary outcome was postoperative pain scores. Secondary outcomes included opioid usage details, adverse events, and blood loss. Exclusion criteria were the non-pediatric population (>21 years old), non-idiopathic scoliosis patients, review articles, studies with unavailable full texts, studies using epidural morphine as the comparison, and studies in non-English languages.

Study Screening

Using a Preferred Reporting Items for Systematic Reviews and Meta-Analyses (PRISMA)-based technique, the title/abstract and full text of each article were systematically screened for inclusion. Using Rayyan, two reviewers (K.D. and S.W.) finished the abstract to full-text screening in an identical fashion [17]. In compliance with the eligibility criteria, a screening process was carried out. In the event of a discrepancy in decisions during the title and abstract screening stage, studies were automatically included and pushed to the full-text screening stage. Any full-text screening conflicts were addressed by a senior author's input (M.N. and W.K.). At each stage of screening, the Kappa (k) statistic was used to report inter-rater agreement. The agreement was categorized as follows: ≤0.20: poor; 0.21-0.40: fair; 0.41-0.60: moderate; 0.61-0.80: substantial; and 0.81-0.99: excellent [18].

Quality Assessment

The Methodological Index for Non-Randomized Studies (MINORS) was used to assess the quality of the publications included in this review [19]. Each evaluation item is rated on a three-point scale, with a minimum grade of 0 and a maximum grade of 2. Non-comparative studies have a maximum score cap of 16, whereas comparative studies have a cap of 24. The evaluation of the literature was conducted individually by two reviewers (K.D. and S.W.) and disagreements in scoring were addressed by discussing with the senior author (W.K.). Categories were determined in a priori fashion. For comparative studies, a score of 0-12 indicated low quality, a score of 13-18 indicated acceptable quality, and a score of 19-24 indicated exceptional quality. For non-comparative studies, a score of 0-5 indicated low quality, a score of 6-10 indicated acceptable quality, and a score of 11-16 indicated exceptional quality.

The Grading of Recommendations, Assessment, Development, and Evaluation (GRADE) approach was used to assess the certainty of evidence for each outcome. For each outcome, ratings for individual domains were categorized as not serious, serious, or very serious, culminating in an overall assessment of the certainty of evidence as high, moderate, low, to very low. Once again, the evaluation of each outcome was conducted by two independent reviewers (K.D. and S.W.), with discrepancies resolved through discussions with the senior author (W.K.).

Data Abstraction and Statistical Analysis

Data abstraction was performed independently and recorded utilizing Google’s Data Suite (Mountain View, CA). Values were randomly and blindly validated by a second reviewer, and disagreements were resolved by the senior author (W.K.). Statistical analysis involved both meta-analytic and non-meta-analytic statistics (i.e., weighted means). All clinical outcomes were analyzed using the random-effects model by reporting either mean differences (MD) and/or standardized mean differences (SMD) for continuous variables and risk ratios (RRs) for dichotomous variables. Missing standard deviations were calculated from the available statistical data, such as the interquartile range, standard error, and sample size [20].

Results

Study Characteristics and Demographics

**Figure 1:**
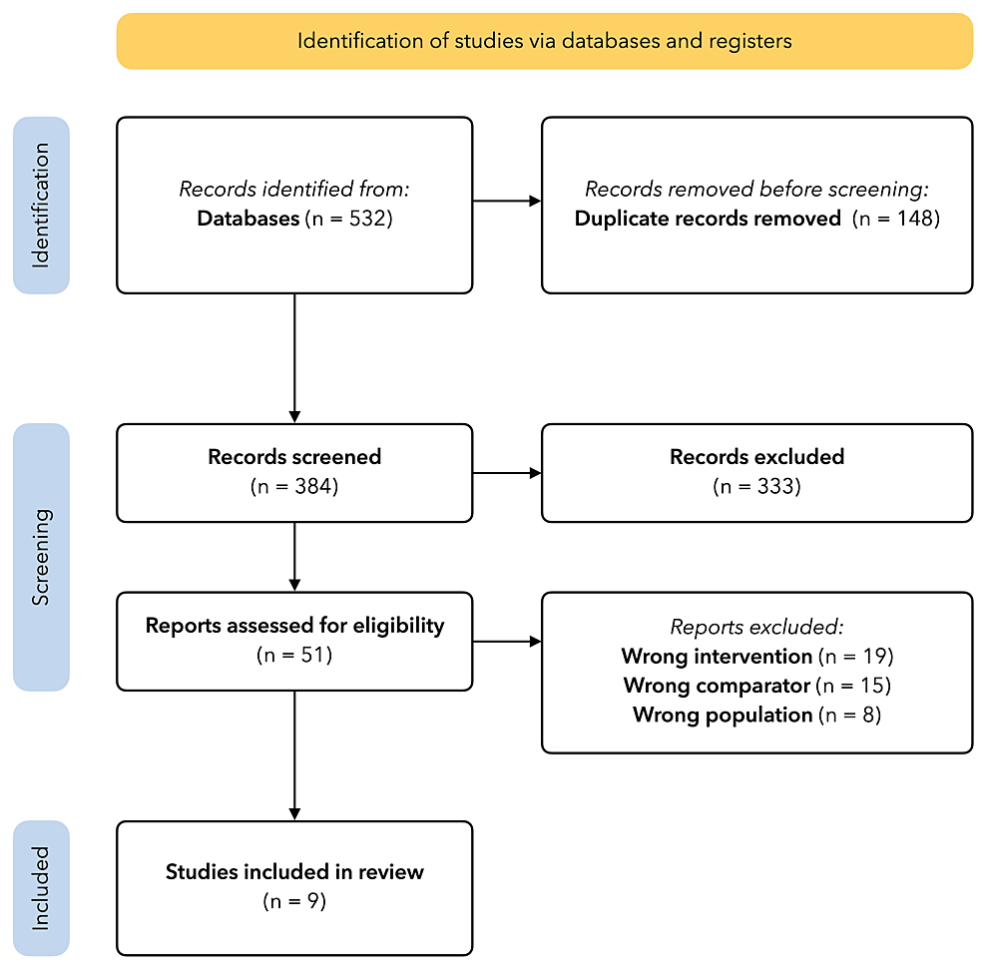
Preferred Reporting Items for Systematic Reviews and Meta-Analyses (PRISMA) study selection diagram

A total of 384 unique articles were identified for screening. Throughout the title/abstract screening and full-text review, substantial to excellent agreement was acquired in both stages, with kappa scores of 0.804 and 0.82, respectively. In the end, nine studies were included in the meta-analysis (Figure 1). This included seven retrospective reviews, one prospective review, and one randomized controlled trial (RCT) (Table 2) [10,14,15,21-26].

**Table 2:** Characteristics of the included studies ITM: intrathecal morphine; n.r.: not reported.

Title	Author	Year published	Study design	Location	# of patients		Mean ITM dosage (µg/kg)
					ITM	Control	
Analgesic effect of low-dose intrathecal morphine after spinal fusion in children	Gall et al. [14]	2001	Randomized control trial	France	20	10	3.5
Intrathecal morphine for postoperative analgesia in patients with idiopathic scoliosis undergoing posterior spinal fusion	Tripi et al. [15]	2008	Retrospective review	USA	339	68	15.36 ± 2.5
A comparison of three methods of pain control for posterior spinal fusions in adolescent idiopathic scoliosis	Milbrandt et al. [22]	2009	Retrospective review	USA	42	41	7
Intrathecal morphine reduces blood loss during idiopathic scoliosis surgery: retrospective study of 256 pediatric cases	Lesniak et al. [23]	2013	Retrospective review	Canada	128	128	11.7 ± 2.4
The safety and efficacy of intrathecal morphine in pediatric spinal deformity surgery: a 25-year single-center experience	Poe-Kochert et al. [21]	2021	Prospective review	USA	578	28	14 ± 2
Zero patient-controlled analgesia is an achievable target for postoperative rapid recovery management of adolescent idiopathic scoliosis patients	Sarwahi et al. [10]	2021	Retrospective review	USA	123	250	1.5
Intrathecal morphine use in adolescent idiopathic scoliosis surgery is associated with decreased opioid use and decreased length of stay	Feltz et al. [24]	2022	Retrospective review	USA	65	40	6
Enhanced recovery following posterior spinal fusion for adolescent idiopathic scoliosis: a medical and economic study in a French private nonprofit pediatric hospital	Jeandel et al. [26]	2023	Retrospective review	France	30	30	n.r.
Utilization of individual components of enhanced recovery after surgery (ERAS) protocol improves post-operative outcomes in adolescent idiopathic scoliosis: a blueprint for progressive adoption of ERAS	Lebel et al. [25]	2023	Retrospective review	Canada	59	81	n.r.

A total of 2060 pediatric patients were included in this review. A total of 1384 patients received ITM (ITM group) while the remaining 676 patients underwent standard pain management (control group). Patients' demographics were similar across both groups (Table 3). For ITM and control groups, mean age (years) was 14.33 ± 1.99 and 14.64 ± 2.08 (n = 8 studies), the mean weight (kg) was 55.28 ± 13.31 and 54.89 ± 14.38 (n = 4 studies), mean BMI was 21.54 ± 4.24 and 21.64 ± 4.53 (n = 3 studies), and females comprise 83.53% and 82.38% of patients (n = 6 studies), respectively. A summary of all clinical outcomes can be found in Table 3.

**Table 3:** Summary of patient demographics, opioid usage, pain scores, adverse events, and intraoperative blood loss ITM: intrathecal morphine; PICU: pediatric intensive care unit; POD: postoperative day; SD: standard deviation; ME: IV morphine equivalent; SMD: standardized mean difference; CI: confidence interval.

Outcome		ITM	Control	# of patients		# of studies	p-value
				ITM	Control		
Patient demographics	Total patients, n	1384	676	1384	676	9	-
	Age (years), mean ± SD	14.33 ± 1.99	14.64 ± 2.08	1319	636	8	-
	Weight (kg), mean ± SD	55.28 ± 13.31	54.89 ± 14.38	1065	234	4	-
	BMI, mean ± SD	21.54 ± 4.24	21.64 ± 4.53	760	359	3	-
	Females, %	83.53	82.38	1269	596	6	-
	Surgery duration (h), mean ± SD	4.96 ± 1.07	4.51 ± 0.84	1065	234	4	-
	# of vertebrae fused, mean ± SD	11.75 ± 1.64	11.67 ± 1.85	980	568	7	-
	Length of stay (days), mean ± SD	4.39 ± 0.84	5.11 ± 0.98	1194	497	6	-
Opioid usage	ITM dosage (µg/kg), mean (range)	12.15 (1.5, >24)	-	1167	437	6	-
	Intraoperative usage (mg/kg ME), mean ± SD	0.33 ± 0.36	1.18 ± 0.62	482	328	3	<0.00001
	Start of opioids postoperation (h), mean ± SD	16.50 ± 4.12	6.60 ± 1.88	937	106	3	<0.00001
	0-24 h usage (mg/kg ME), mean ± SD	1.18 ± 0.55	2.92 ± 0.80	244	382	4	0.03
	0-48 h usage (mg/kg ME), mean ± SD	1.68 ± 0.53	4.25 ± 1.17	521	399	3	0.05
	Total usage reported, SMD (95% CI)	-2.10 (-3.48, -0.73)	2.10 (3.48, 0.73)	648	490	6	0.003
Pain scores	POD 0 (Wong-Baker score), mean ± SD	0.83 ± 1.21	4.46 ± 2.15	679	150	3	0.0009
	POD 1 (Wong-Baker score), mean ± SD	2.74 ± 1.86	4.06 ± 1.88	709	180	4	0.17
	POD 2 (Wong-Baker score), mean ± SD	3.97 ± 1.38	4.67 ± 1.68	101	122	2	0.002
	POD 3 (Wong-Baker score), mean ± SD	4.23 ± 1.5	4.97 ± 2	89	111	2	0.04
	Mean pain score, SMD (95% CI)	-1.30 (-2.29, -0.31)	1.30 (2.29, 0.31)	1171	498	6	0.01
Adverse events	Nausea/vomiting, %	25.77	37.86	1040	346	3	0.36
	Pruritus, %	7.3	3.88	1082	387	4	0.67
	Respiratory depression, %	2.59	0.52	1082	387	4	0.34
	PICU admission, %	3.05	0	917	96	2	0.29
Blood loss	Total blood loss (L), mean ± SD	0.774 ± 0.524	1.740 ± 0.963	726	166	3	0.0001
	Blood loss (ml/kg), mean ± SD	14.08 ± 9.49	32.28 ± 19.29	726	166	3	0.02
	Blood loss (ml/segment fusion), mean ± SD	65.42 ± 45.01	140.29 ± 78.85	726	166	3	0.0002

Quality Assessment

The MINORS score for the comparative non-RCT studies was 18.2, indicating moderate quality studies. The quality of evidence for each outcome is summarized in Table 4. The quality of evidence varied for pain scores; being moderate for postoperative day (POD) 2; low for POD 0, POD 3, and mean overall pain scores; and very low for POD 1. The low and very low quality of evidence was due to the subjective nature of determining pain scores and inconsistency between studies. The quality of evidence for opioid usage also varied, being high for the time to first request for opioids, moderate for intraoperative opioid usage and total opioid usage, and low for 24 hours and 48 hours usage. The low quality of evidence was due to inconsistency between studies. The quality of evidence for adverse events was low to very low; being low for nausea/vomiting incidence and pediatric intensive care unit (PICU) admissions; and very low for the incidence of pruritus and respiratory depression. This was due to the lack of any statistically significant difference between ITM and control groups and inconsistency between studies. Lastly, the quality of evidence for blood loss was moderate.

**Table 4:** Certainty of evidence for each outcome using the Grading of Recommendations, Assessment, Development, and Evaluation (GRADE) approach a = high heterogeneity, some confidence intervals do not overlap. b = scoring is subjective. c = large confidence intervals. ITM: intrathecal morphine; SMD: standardized mean difference; POD: postoperative day; PICU: pediatric intensive care unit; MD: mean difference.

Outcome	Certainty assessment							# of patients		Effect		Certainty	Importance
	# of studies	Study design	Risk of bias	Inconsistency	Indirectness	Imprecision	Other considerations	ITM	Control	Relative (95% CI)	Absolute (95% CI)		
Mean pain scores (SMD)	6	Observational studies	Not serious	Serious^a^	Not serious	Serious^b^	Very strong association	1171	498	-	SMD 1.3 SD lower (2.29 lower to 0.31 lower)	Low	CRITICAL
POD 0 (Wong-Baker pain scores)	3	Observational studies	Not serious	Serious^a^	Not serious	Serious^b^	Very strong association	679	150	-	MD 2.46 lower (3.91 lower to 1.01 lower)	Low	CRITICAL
POD 1 (Wong-Baker pain scores)	4	Observational studies	Not serious	Not serious	Not serious	Serious^b^	None	709	180	-	MD 0.86 lower (2.09 lower to 0.37 higher)	Very low	CRITICAL
POD 2 (Wong-Baker pain scores)	2	Observational studies	Not serious	Not serious	Not serious	Serious^b^	Very strong association	101	122	-	MD 0.64 lower (1.05 lower to 0.24 lower)	Moderate	CRITICAL
POD 3 (Wong-Baker pain scores)	2	Observational studies	Not serious	Not serious	Not serious	Serious^b^	Strong association	89	111	-	MD 0.82 lower (1.59 lower to 0.05 lower)	Low	CRITICAL
Intraoperative opioid usage	3	Observational studies	Not serious	Serious^a,c^	Not serious	Not serious	Very strong association	482	328	-	MD 0.71 lower (0.99 lower to 0.44 lower)	Moderate	IMPORTANT
Time to starting opioids postoperation	3	Observational studies	Not serious	Not serious	Not serious	Not serious	Very strong association	937	106	-	MD 9.62 higher (8.13 higher to 11.11 higher)	High	IMPORTANT
0-24 h opioid usage	4	Observational studies	Not serious	Serious^a^	Not serious	Not serious	Strong association	244	382	-	MD 1.23 lower (2.33 lower to 0.14 lower)	Low	CRITICAL
0-48 h opioid usage	3	Observational studies	Not serious	Serious^a^	Not serious	Not serious	Strong association	521	399	-	MD 1.94 lower (3.86 lower to 0.01 lower)	Low	CRITICAL
Total opioid usage reported (SMD)	6	Observational studies	Not serious	Serious^a^	Not serious	Not serious	Very strong association	648	490	-	SMD 2.1 SD lower (3.48 lower to 0.73 lower)	Moderate	CRITICAL
Nausea/vomiting	3	Observational studies	Not serious	Not serious	Not serious	Not serious	None	268/1040 (25.8%)	131/346 (37.9%)	RR 0.91 (0.73 to 1.12)	34 fewer per 1,000 (from 102 fewer to 45 more)	Low	IMPORTANT
Pruritis	4	Observational studies	Not serious	Serious^a^	Not serious	Not serious	None	79/1082 (7.3%)	15/387 (3.9%)	RR 1.24 (0.47 to 3.28)	9 more per 1,000 (from 21 fewer to 88 more)	Very low	IMPORTANT
Respiratory depression	4	Observational studies	Not serious	Serious^a^	Not serious	Not serious	None	28/1082 (2.6%)	2/387 (0.5%)	RR 1.91 (0.51 to 7.15)	5 more per 1,000 (from 3 fewer to 32 more)	Very low	CRITICAL
PICU admission	2	Observational studies	Not serious	Not serious	Not serious	Not serious	None	28/917 (3.1%)	0/96 (0.0%)	RR 2.91 (0.40 to 21.12)	0 fewer per 1,000 (from 0 fewer to 0 fewer)	Low	CRITICAL
Blood loss	3	Observational studies	Not serious	Not serious	Not serious	Serious^c^	Very strong association	726	166	-	MD 883.57 SD lower (1337.51 lower to 429.64 lower)	Moderate	CRITICAL

Pain Scores

Although postoperative pain scores were reported by seven studies, Gall et al. (2001) reported their pain scores using a box plot that prevented accurate abstraction [14]. Of the remaining six studies, five used the Wong-Baker scale [15,21,22,25,26], in which physicians used the patient's face to estimate their level of pain, and one study used the Visual Analog Scale (VAS) [10], in which the pain score was reported by the patient. Moreover, some studies reported mean overall pain scores, and others reported pain scores on various PODs, from which the mean pain score was calculated.

**Figure 2:**
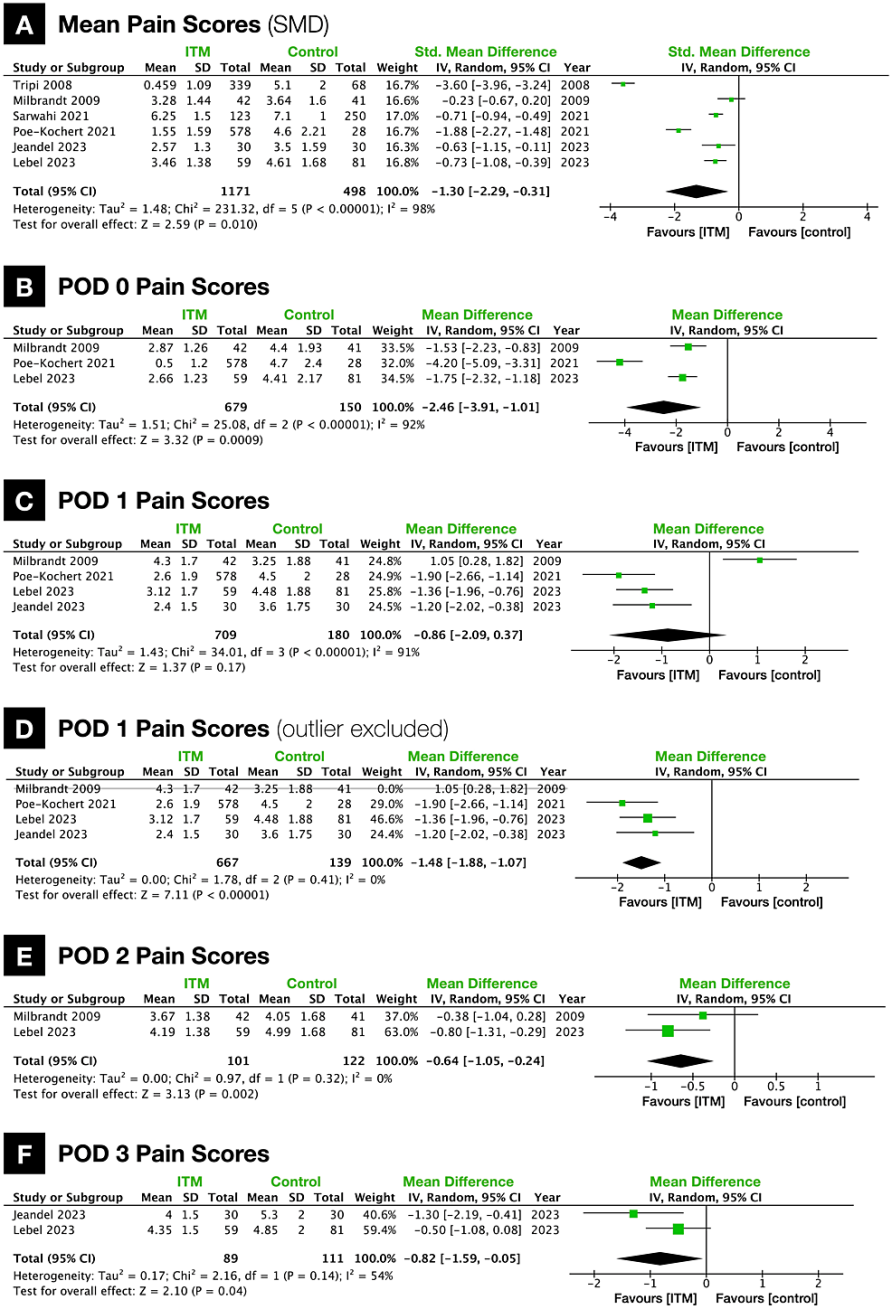
Forest plots of pain scores (A) Mean pain scores (Wong-Baker and Visual Analog Scale combined, SMD). (B) Wong-Baker pain scores on postoperative day (POD) 0. (C) Wong-Baker pain scores on POD 1. (D) Wong-Baker pain scores on POD 1, excluding data from Milbrandt et al. (2009) due to its classification as an outlier. (E) Wong-Baker pain scores on POD 2. (F) Wong-Baker pain scores on POD 3. ITM: intrathecal morphine; SMD: standardized mean difference.

When the mean pain scores of all six studies were pooled, it was significantly lower in ITM patients (SMD: -1.30 {-2.29, -0.31}; p = 0.01; heterogeneity, I2 = 98%; n = 6 studies; Figure 2A). Additionally, pain scores were significantly lower in ITM patients on POD 0 (ITM vs. control, 0.83 vs. 4.46; MD: -2.46 {-3.91, -1.01}; p = 0.0009; heterogeneity, I2 = 92%; n = 3 studies; Figure 2B), POD 2 (ITM vs. control, 3.97 vs. 4.67; MD: -0.64 {-1.05, -0.24}; p = 0.002; heterogeneity, I2 = 0%; n = 2 studies; Figure 2E), and POD 3 (ITM vs. control, 4.23 vs. 4.97; MD: -0.82 {-1.59, -0.05}; p = 0.04; heterogeneity, I2 = 54%; n = 2 studies; Figure 2F). While there was no significant difference in pain scores on POD 1 (ITM vs. control, 2.74 vs. 4.06; MD: -0.86 {-2.09, 0.37}; p = 0.17; heterogeneity, I2 = 91%; n = 4 studies; Figure 2C), following the exclusion of one study identified as an outlier, pain scores were significantly lower in ITM patients (ITM vs. control, 2.64 vs. 4.29; MD: -1.48 {-1.88, -1.07}; p < 0.00001; heterogeneity, I2 = 0%; n = 3 studies; Figure 2D).

Opioid Usage

Three studies reported intraoperative opioid usage and six studies reported postoperative opioid usage in some form. Among the latter, three studies reported 0-24 hours usage and three studies reported 0-48 hours usage. Additionally, Gall et al. (2001) did graphically report opioid usage for the first 18 hours, and due to the near-linear relationship, the predicted 0-24 hours usage was also included in the analysis [14]. To standardize opioid usage (when possible), an opioid conversion table from Admiraal et al. (2021) was used to calculate usage as IV morphine equivalents (ME) in mg/kg [27].

**Figure 3:**
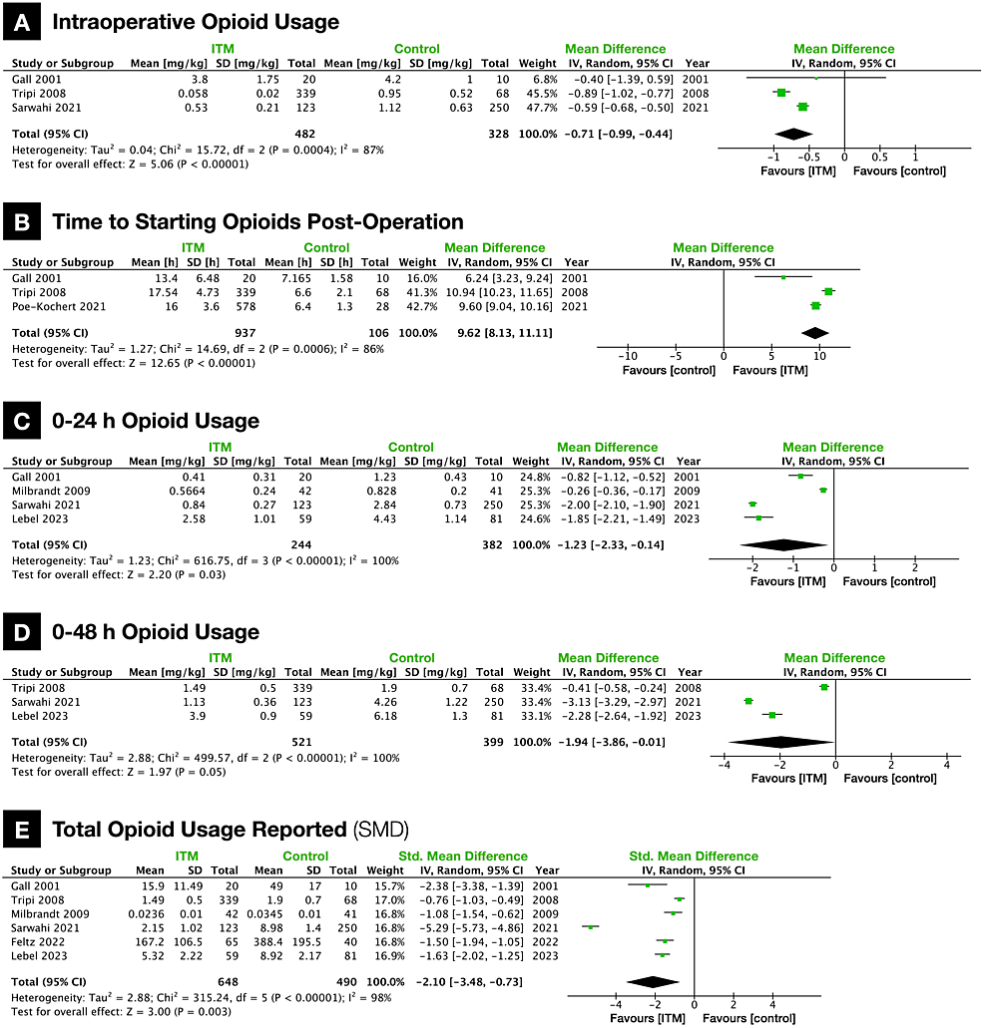
Forest plots of opioid usage (A) Intraoperative opioid usage (mg/kg IV morphine equivalents). (B) Time to start opioids postoperation (h). (C) Opioid usage in the first 24 hours (mg/kg IV morphine equivalents). (D) Opioid usage in the first 48 hours (mg/kg IV morphine equivalents). (E) Total opioid usage reported (SMD). ITM: intrathecal morphine; SMD: standardized mean difference.

Intraoperative opioid usage was significantly lower in ITM patients (ITM vs. control, 0.33 vs. 1.18 mg/kg ME; MD: -0.71 {-0.99, -0.44}; p < 0.00001; heterogeneity, I2 = 95%; n = 3 studies; Figure 3A). Following surgery, the time to first request for opioids was significantly longer in ITM patients (ITM vs. control, 16.50 vs. 6.60 hours; MD: 9.62 {8.13, 11.11}; p < 0.00001; heterogeneity, I2 = 86%; n = 3 studies; Figure 3B). Mean opioid usage was significantly lower in ITM patients 24 hours postoperation (ITM vs. control, 1.18 vs. 2.92 mg/kg ME; MD: -1.23 {-2.33, -0.14}; p = 0.03; heterogeneity, I2 = 100%; n = 4 studies; Figure 3C) and 48 hours postoperation (ITM vs. control, 1.68 vs. 4.25 mg/kg ME; MD: -1.94 {-3.86, -0.01}; p = 0.05; heterogeneity, I2 = 100%; n = 3 studies; Figure 3D). When pooling the final opioid usage reported from all six studies (without ME conversion and not matched for a time period), there was significantly lower usage in the ITM group (SMD: -2.10 {-3.48, -0.73}; p = 0.003; heterogeneity, I2 = 98%; n = 6 studies; Figure 3E).

Adverse Events

Commonly reported complications included nausea/vomiting (n = 5 studies), pruritus (n = 5 studies), and respiratory depression (n = 5 studies). PICU admissions were also reported (n = 2 studies); however, these may not always be solely due to adverse events, as differences in institutional practices could contribute to varying admission rates. Two studies were excluded from the analysis because their data could not be accurately pooled with the other studies. Unlike the other studies, Gall et al. (2001) reported complications as the total number of events and not the number of patients [14], and Milbrandt et al. (2009) reported nausea and vomiting separately [22].

**Figure 4:**
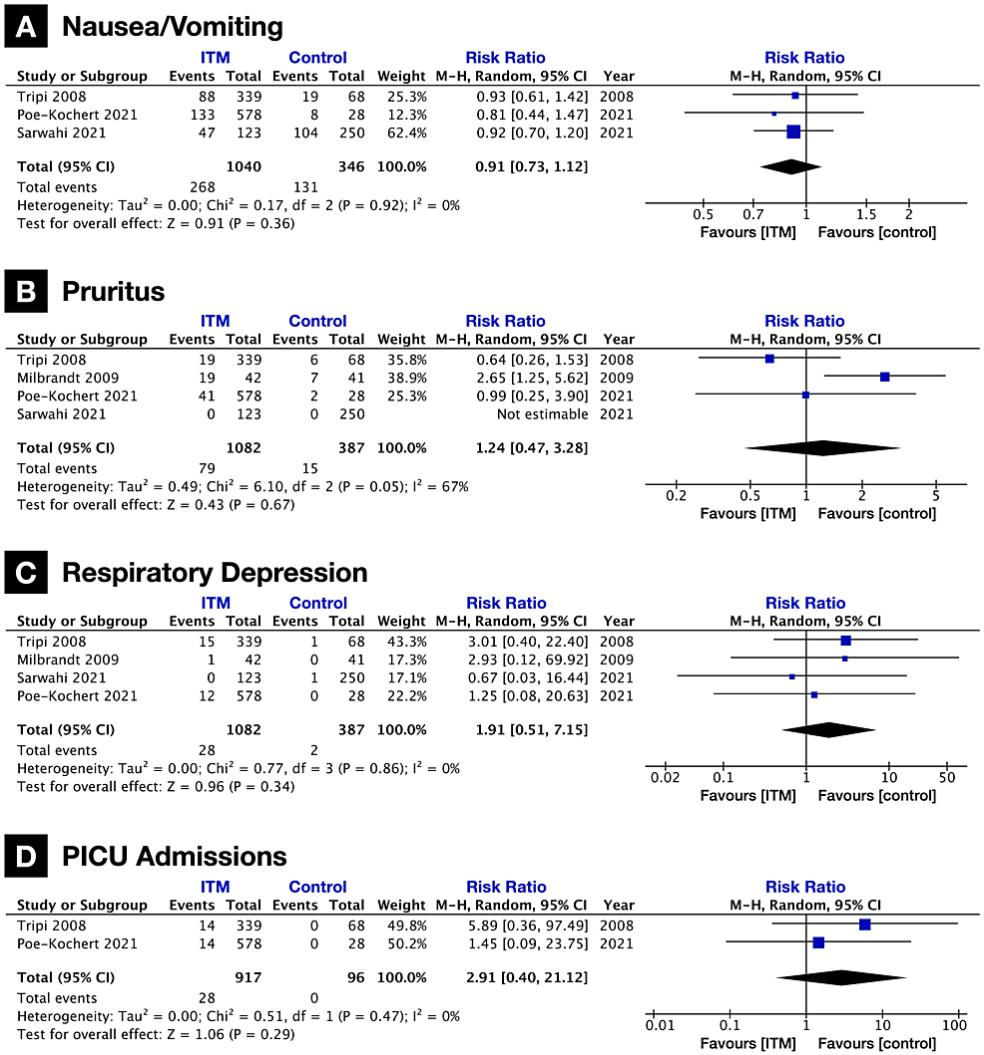
Forest plots of adverse events (A) Nausea and vomiting. (B) Pruritus. (C) Respiratory depression. (D) PICU admissions. ITM: intrathecal morphine; SMD: standardized mean difference; PICU: pediatric intensive care unit.

A meta-analysis revealed no statistically significant difference in the risk of these adverse events. The incidence and risk ratios of each were as follows: nausea/vomiting (ITM vs. control, 25.8% vs. 37.9%; RR, 0.91 {0.73, 1.12}; p = 0.36; heterogeneity, I2 = 0%; n = 3 studies; Figure 4A), pruritus (ITM vs. control, 7.3% vs. 3.9%; RR, 1.24 {0.47, 3.28}; p = 0.67; heterogeneity, I2 = 67%; n = 4 studies; Figure 4B), respiratory depression (ITM vs. control, 2.6% vs. 0.5%; RR, 1.91 {0.51, 7.15}; p = 0.34; heterogeneity, I2 = 0%; n = 4 studies; Figure 4C), and PICU admissions (ITM vs. control, 3.1% vs. 0.0%; RR, 2.91 {0.40, 21.12}; p = 0.29; heterogeneity, I2 = 0%; n = 2 studies; Figure 4D).

Intraoperative Blood Loss

**Figure 5:**
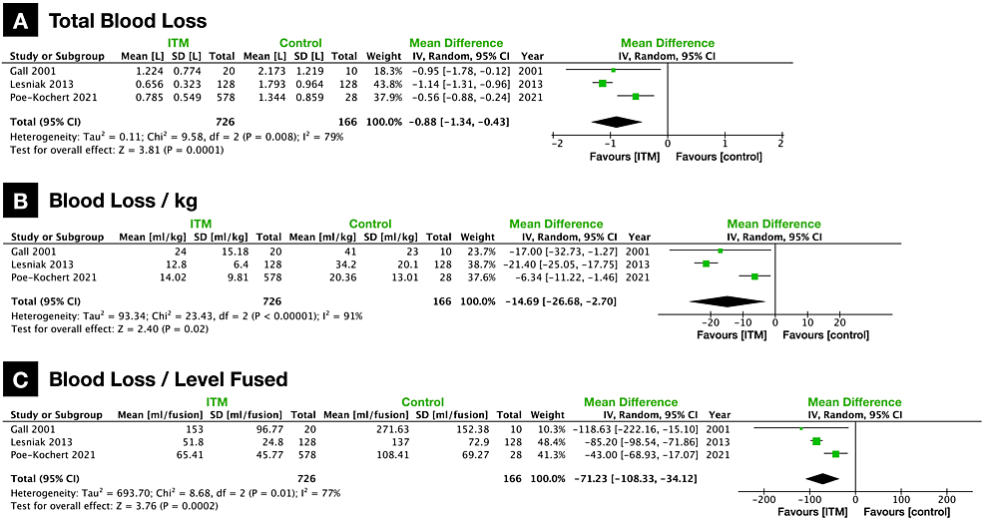
Forest plots of intraoperative blood loss (A) Total intraoperative blood loss (L). (B) Intraoperative blood loss per kilogram body weight (ml/kg). (C) Intraoperative blood loss per number of levels fused (ml/fusion). ITM: intrathecal morphine.

Intraoperative blood loss was reported in three studies. In these studies, ITM was administered during the initial stages, following the induction of anesthesia but preceding the spinal fusion procedure [14,21,23]. Total blood volume lost was significantly lower in the ITM group (ITM vs. control, 0.774 vs. 1.740 L; MD: -0.88 {-1.34, -0.43}; p = 0.0001; heterogeneity, I2 = 79%; n = 3 studies; Figure 5A). Blood loss was also significantly lower when accounting for patient weight (ITM vs. control, 14.1 vs. 32.3 ml/kg; MD: -14.69 {-26.68, -2.70}; p = 0.02; heterogeneity, I2 = 91%; n = 3 studies; Figure 5B) and levels fused during spine surgery (ITM vs. control, 65.4 vs. 140.3 ml/level fused; MD: -71.23 {-108.33, -34.12}; p = 0.0002; heterogeneity, I2 = 77%; n = 3 studies; Figure 5C).

Discussion

In this study, we sought to analyze the effect of ITM administration during posterior spinal fusion by comparing it to standard pain management (intravenous opioids only). In both groups, intravenous opioids were used intraoperatively and postoperatively for analgesia. In this meta-analysis, we compared the clinical outcomes of 1295 ITM and 565 control pediatric patients.

Our analysis reveals ITM patients had significantly lower pain scores (GRADE evaluation, moderate to very low). As a result, the time to start opioids after surgery was also significantly delayed in the ITM group (GRADE evaluation, high), indicating a longer duration of analgesia associated with ITM. Studies have estimated that its analgesic effect lasts for at least 12 to 18.8 hours [2]. Furthermore, these lower pain scores were achieved using significantly lower doses of opioids, both intraoperatively (GRADE evaluation, moderate) and postoperatively (GRADE evaluation, moderate to low).

Techniques to minimize blood loss in spinal fusions are critical given the morbidity associated with excess blood loss [28]. Two factors known to predict intraoperative blood loss are the duration of surgery and the number of levels fused [23]. Our meta-analysis shows that intraoperative blood loss was significantly lower in the ITM group, despite the duration of surgery, levels fused, and mean weight being similar across both groups (GRADE evaluation, moderate).

The current leading hypothesis to explain this effect of ITM is that it may reduce mean arterial pressure during surgery, which subsequently reduces intraoperative blood loss [14,23,29]. Although the mechanism is not entirely understood, our findings strongly suggest that the observed decrease in blood loss can be attributed to ITM. In all three studies reporting on blood loss, ITM was administered earlier in the procedure before the spinal fusion, implicating it was likely responsible for reducing blood loss during surgery. Following a more detailed analysis, the RCT by Gall et al. had two ITM dosage groups (2 µg/kg and 5 µg/kg), and they reported a dose-dependent reduction in intraoperative blood loss [14]. They also noted there was a lower sufentanil and propofol requirement in ITM patients to achieve a similar level of hypotension to control patients [14]. Considering this was a double-blind study, this is strong evidence supporting the ability of ITM to significantly lower intraoperative blood loss. Adding to this, all surgeries in the study by Lesniak et al. were performed by the same surgeon and anesthesiology team, largely eliminating differences in surgical and anesthetic technique as a factor contributing to variances in blood loss [23]. Like Gall et al., they also concluded that the significant reduction in blood loss was a result of ITM. Lesniak et al. further stated that the number of blood transfusions and the volume of blood transfused were both significantly lower in ITM patients [23]. Given that blood transfusions are associated with several risks, the substantial reduction in intraoperative blood loss is a notable advantage to ITM [23]. An important consideration for future studies is whether the standard of care in different settings included antifibrinolytics. If the timeline for ITM and antifibrinolytics initiation overlapped, this may skew findings related to the sole impact of ITM.

There are concerns about adverse events associated with ITM use. Although our analysis showed that there was no statistically significant difference in the incidence of adverse events between ITM and control, ITM patients did have a lower incidence of nausea/vomiting (GRADE evaluation, low), and a higher incidence of pruritus (GRADE evaluation, very low), respiratory depression (GRADE evaluation, very low), and PICU admissions (GRADE evaluation, low). Given these findings, it is important to note that nausea and vomiting, as well as pruritus, are relatively minor complications, whereas respiratory depression and PICU admission are the primary concerns surrounding ITM use. It is also worth mentioning that PICU admission is not always classified as a complication, as it is part of the standard protocol in certain institutions.

Continuing this exploration, it is important to interpret the available data within the context of the ITM dose administered. Although there is a lack of consensus regarding optimal ITM dosage, some studies indicate that administering low doses ranging from 1.5 to 5 µg/kg provides improved analgesia versus control, as well as minimal risk of complications in pediatric patients [10,14,30]. Despite the challenges posed by the limited number of studies in our review, we attempted to analyze the available data as best as possible.

In the present study, ITM was administered at doses ranging from 1.5 µg/kg [10] to over 24 µg/kg [15]. We classified the studies into three pools: low dose, which includes Milbrandt et al. (2009) and Sarwahi et al. (2021) (dose: 1.5-7 µg/kg, mean: 2.9 µg/kg, n = 164 patients); moderate dose, which includes Poe-Kochert et al. (2021) and the first cohort from Tripi et al. (2008) (dose: 9-19 µg/kg, mean: 14.0 µg/kg, n = 871 patients); and high dose, which includes the second cohort from Tripi et al. (2008) (dose: >19 µg/kg, mean: 24 µg/kg, n = 46 patients) [10,15,21,22]. PICU admissions were reported by the latter two pools, in which the moderate dose pool had a much lower PICU admission rate than the high dose pool (2.30% vs. 17.4%, respectively). Among both pools, it is worth mentioning there were no PICU admissions in the control groups (n = 96 patients). However, this discrepancy may be due to a sampling error as there were 10 times more patients in the ITM group. Furthermore, the lack of blindness may have led to increased caution for ITM patients.

Upon further examination, the primary cause for PICU admission among ITM patients was respiratory depression, accounting for 21 of the 28 total admissions. Examining the effect of ITM dosage on the rate of respiratory depression, both the low dose and moderate dose pools had a low incidence (0.61% vs. 2.30%, respectively) that was similar to the control patients (0.52%, n = 387). The high dose pool, on the other hand, had a much greater incidence (15.2%), indicating a dose greater than >19 µg/kg has high risk, aligning with earlier studies that have suggested ITM-related respiratory depression may be dose-dependent [10]. Among all nine studies (n = 1384 ITM patients), there were no serious cases of respiratory depression and no re-intubations were required for any ITM patient as all events were temporary with no permanent side effects [10,14,15,21-26]. Of the cases, 10-14 were resolved with naloxone alone while the rest required no further intervention and were resolved only with observation [15,21]. The RCT by Gall et al. (2001) also stated their cases of respiratory depression required no intervention [14]. Considering all cases of respiratory depression were minor, there is a low risk and high reward associated with low to moderate doses of ITM.

More studies are warranted to identify which dose maximizes analgesia and the other benefits of ITM and minimizes the risk of complications. Although our analysis indicates that a low dose of ITM causes virtually no increase in the incidence of respiratory depression, and a moderate dose only marginally increases it, additional data comparing pain relief, opioid consumption, and blood loss across these dosage groups is required. High-quality prospective studies, modeled after the approach taken by Tripi et al. (2008), are essential to pinpoint this optimal dose [15]. In this retrospective review, patients were divided into three groups: control, moderate dose of ITM, and high dose of ITM [15]. Future studies are encouraged to follow a similar methodology, with an emphasis on low and moderate dosage groups to determine which is more optimal.

Lastly, there are limitations to our study. Given the variability in ITM dosing across the studies, our results may not accurately reflect the ideal and most effective ITM dosage. The efficacy and spread of ITM along the neuraxis are influenced by several factors, including the location and timing of administration, rate of injection, and concentration of the injectate. As we had insufficient data, a thorough analysis of these parameters was not possible. Similarly, for pain scores and opioid usage, there was inconsistent reporting of data, complicating direct comparisons. When looking at opioid usage, the types and doses of opioids used were not reported consistently. The control protocol was also inconsistently reported and generally followed the institutional protocols of the reporting site. Therefore, the non-ITM controls were not standardized. Additionally, there was only one RCT that was included, and the lack of blindness may have yielded greater precautionary measures for ITM patients. Furthermore, the criteria for admitting patients to the PICU after spinal fusion surgery differ among institutions, with some treating it as a more routine procedure than others.

## Conclusions

In conclusion, this meta-analysis demonstrates a superior analgesic effect and a low complication profile when using ITM in conjunction with standard intravenous opiates. While high doses of ITM were associated with increased rates of respiratory depression, low to moderate doses had a complication profile similar to control while providing better analgesia. Additionally, ITM significantly reduced hospital stay, intraoperative and postoperative opioid usage, and intraoperative blood loss. Although further comparative studies are warranted in elucidating optimal dosing and complication profile, ITM appears to be a safe and powerful tool in the clinician’s armamentarium of multimodal postoperative analgesia.
